# Demographics of childhood hypertension in the UK: a report from the Southeast England

**DOI:** 10.1038/s41371-022-00732-7

**Published:** 2022-08-06

**Authors:** Emily Haseler, Cheentan Singh, Joanna Newton, Nabil Melhem, Manish D. Sinha

**Affiliations:** 1 Department of Paediatric Nephrology, Evelina London Children’s Hospital, Guys & St Thomas’ NHS Foundation Trust, Westminster Bridge Road, London, SE1 7EH UK; 2 North Middlesex Hospital, London, UK; 3 Kings College London, London, UK

**Keywords:** Hypertension, Risk factors, Diagnosis

## Abstract

We aimed to describe hypertensive phenotype and demographic characteristics in children and adolescents referred to our paediatric hypertension service. We compared age, ethnicity and BMI in primary hypertension (PH) compared to those with secondary hypertension (SH) and white coat hypertension (WCH). Demographic and anthropometric data were collected for children and adolescents up to age 18 referred to our service for evaluation of suspected hypertension over a 6 year period. Office blood pressure (BP) and out of office BP were performed. Patients were categorised as normotensive (normal office and out of office BP), WCH (abnormal office BP, normal out of office BP), PH (both office and out of office BP abnormal, no underlying cause identified) and SH (both office and out of office BP abnormal, with a secondary cause identified). 548 children and adolescents with mean ± SD age of 10.1 ± 5.8 years and 58.2% girls. Fifty seven percent (*n* = 314) were hypertensive; of these, 47 (15%), 84 (27%) and 183 (58%) had WCH, PH and SH, respectively. SH presented throughout childhood, whereas PH and WCH peaked in adolescence. Non-White ethnicity was more prevalent within those diagnosed with PH than both the background population and those diagnosed with SH. Higher BMI *z*-scores were observed in those with PH compared to SH. Hypertensive children <6 years are most likely to have SH and have negligible rates of WCH and PH. PH accounted for 27% of hypertension diagnoses in children and adolescents, with the highest prevalence in adolescence, those of non-White Ethnicity and with excess weight.

## Introduction

Arterial hypertension is one of the most prevalent chronic health concerns in adults; half of ischaemic heart disease and one third of strokes are thought to be secondary to hypertension [1]. In children and adolescents, hypertension is associated with target organ damage during childhood, increased risk of hypertension as a young adult and adverse cardiovascular outcomes in adulthood [2–4]. The increasing prevalence of primary hypertension in children and adolescents is largely due to the increasing prevalence of childhood obesity [5–7]. There are no recent clinical studies describing the current demographic profile of childhood hypertension within the UK. In this brief report, we describe the demographic characteristics of children and adolescents referred for assessment of elevated blood pressure (BP) to our regional Paediatric hypertension service.

## Methods

### Study population

The Paediatric Hypertension service at the Evelina London Children’s Hospital (ELCH) receives referrals from London and the Southeast of England covering a population of approximately 1.7 million children and adolescents. The referral population in London has a more diverse ethnic makeup than the wider UK population, in particular a large Black African heritage population [8]. Referrals to the hypertension service are received from Primary Care (General Practitioners), Secondary care (e.g., district general hospitals or community based paediatric services) and Tertiary Care services. This was a retrospective analysis evaluating results of clinical investigations. No consent from patients was indicated and ethical approval was not required.

### Inclusion and exclusion criteria

All children and adolescents referred to the hypertension service between January 2013–December 2018 were entered to a prospectively maintained electronic database which was updated regularly following completion of assessments. The inclusion criteria were (i) age ≤18 years; (ii) completion of initial assessment of hypertension status including office BP (iii) out of office BP measurement. Exclusion criteria were: (i) patients receiving dialysis; (ii) failure to attend clinic for assessment following referral; (iii) incomplete investigations or unable to tolerate investigations to assign a diagnosis (e.g., transfer to adult services before initial workup investigations could be completed).

### Data collection

We collected clinical and demographic characteristics including: (i) age at the time of initial assessment; (ii) sex; (iii) patient assigned ethnicity; (iv) referral source (primary, secondary or tertiary care); (v) height, weight, body mass index (BMI); (vi) office BP; (vii) out-of-office BP measurement technique and outcome following assessment: normotension, white coat hypertension (WCH), and confirmed hypertension; (vii) cause of hypertension, Primary Hypertension (PH) or Secondary Hypertension (SH); and (viii) underlying cause (e.g., renal disease, cardiac disease or other) if SH was recorded.

For children under 2, BMI was categorised according to age- and sex specific criteria of the World Health Organisation (WHO): overweight 91st to 98th centile (+2 < >BMI SDS ≤ + 3), and obesity ≥ 98th centile (BMI SDS > + 3) [9–11]. For 2–15 year olds we used cut-off values from the International Obesity Task Force (IOTF) to categorise their BMI [12, 13]. These age and sex-specific cut-off values are based on percentile curves passing through adult health related cut-off points for overweight (BMI of 25 kg/m^2^), and obesity (BMI of 30 kg/m^2^) at the age of 18 years. Both overweight and obese status were categorised as excess weight.

### Measurement and interpretation of Office BP

Office BP was measured on the right arm by a trained observer using a calibrated aneroid sphygmomanometer (Welch Allyn Dura-Shock DS54 or WelchAllyn DuraShock DS-66 trigger model, Welch Allyn New York, New York, USA) and auscultation according to British Hypertension Society guidelines. BP measurement devices in clinical use were checked once every 1–2 years for accuracy. The mean of three values of systolic BP (SBP) and diastolic BP (DBP) were used for analyses. We defined hypertension as office BP above the 95th percentile threshold for sex, age and height measured as per the 2016 European Society of Hypertension (ESH) guidelines in 1–17 year old patients [6]. In those aged <1 year we used the Second Task Force normative values defining normal BP below the 95th percentile [14]. Patients were also categorised as being hypertensive if they were on anti-hypertensive medication at the time of initial review.

### Measurement and interpretation of out-of-office BP

24-h ambulatory blood pressure monitoring (ABPM) was performed where possible as the gold standard for out-of-office BP assessment. In those younger than 5-years of age or unable to tolerate ABPM, we used home BP measurements. We have previously reported the results of an alternative method to measure systolic BP in such children by parents using at *h*ome a hand-held *d*oppler device and aneroid sphygmomanometer for systolic *b*lood *p*ressure *m*easurement (HDBPM) [15, 16]. HDBPM was only offered to those with systolic or systo-diastolic hypertension when measured in office. For those unable to tolerate ABPM or perform HDBPM, blood pressure was measured at home by community based health care professionals.

**Fig. 1:**
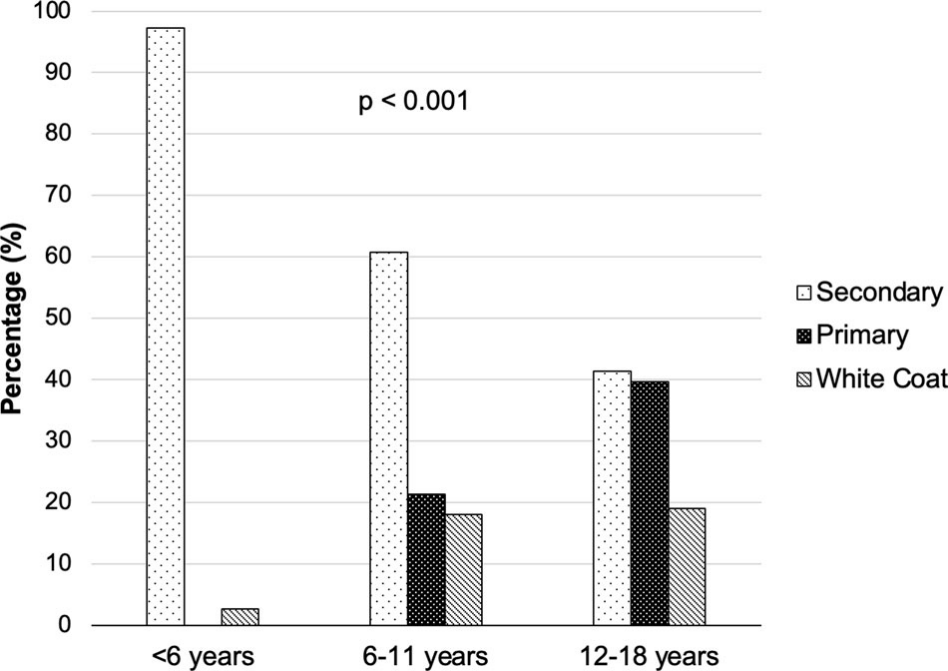
Prevalence of primary, secondary and white coat hypetension by age. Data arranged by age tertile and pertains to those aged <18 years of age diagnosed with hypertension.

Further details for each out-of-office BP monitoring technique are shown: (i) 24-h ABPM was performed in children using the Spacelabs Healthcare 90217 recorder (OSI Systems, Hawthorne, California) and an appropriately sized cuff. We defined ambulatory hypertension as per the 2016 ESH guidelines; [6] (ii) HDBPM: In those who underwent HDBPM, following auscultatory office BP measurement, systolic blood pressure was measured thrice in quick succession by the health professional using a hand-held doppler device and the same aneroid sphygmomanometer and cuff. The mean of three BP values was used. Parents then underwent a 20–30 min education session with a health professional in order to learn the technique until the health professional was satisfied with their proficiency. Parents were asked to record three consecutive BP readings twice daily for 2 weeks, ideally morning and evening around similar times. Further detail on this technique has been published previously; [15, 16] and (iii) Home BP measurement by community nursing professionals: Community nurses visited the family to measure BP. Hypertension was defined as BP > 95th percentile for age, sex and height using the same normative reference values as for office BP measurement for both HDBPM and BP measured at home by health professional. A summary of out of office BP monitoring modalities and their selection is shown in Fig. 1A (supplementary information).

### Statistical analysis

We analysed age and sex distribution of normotensive and those with hypertension by age tertiles (<6, 6–11, 12–18 years). Differences in means between continuous variables were assessed for significance using one way analysis of variance (ANOVA) with Tukey’s post hoc test. Chi-squared test was used for differences in total count between categorical variables. Chi-squared goodness of fit test was used to assess for differences in ethnicity distribution compared to the background population, as derived from ONS census data for South East England from 2011 [8]. SPSS statistics software (IBM Corp. Released 2020. IBM SPSS Statistics for Macintosh, Version 27.0. Armonk, NY: IBM Corp) was used to analyse data. *P* < 0.05 was used to determine statistical significance.

## Results

Over a 6-year period, 548 children and adolescents were evaluated by our service. These included 58.2% (*n* = 319) girls and mean ± SD age at assessment was 10.1 ± 5.8 years. Of the 517 (94.3%) patients for whom ethnicity was recorded, 67.5% (*n* = 349), 19.3% (*n* = 99) and 8.5% (*n* = 44) patients were White, Black-African and South-East Asian ethnic origin respectively. Primary, secondary and tertiary care referrals were 2.6% (*n* = 14), 48.0% (*n* = 263) and 49.4% (*n* = 271). Ambulatory BP monitoring was performed in 57% (*n* = 312), HDBPM in 30% (*n* = 165) and others evaluated in the community. There were *n* = 128 children <6-years old who received HDBPM. Following out of office BP assessment, 42.7% (*n* = 234) children and adolescents were found to be normotensive and hypertension diagnosed in 57.3% (*n* = 314), of whom 57.6% (*n* = 181) were on antihypertensive therapy at the time of assessment.

Demographic and clinical characteristics for hypertensive children and adolescents are shown in Table 1. There was no significant difference between proportions of males and females between the hypertensive groups (*p* = 0.16). Children <6 years were significantly more likely to have normotension following evaluation using appropriate out-of-office monitoring, when compared to children ≥6 years (*p* < 0.001).

**Table 1:** Demographic and clinical characteristics of *n* = 314 children and young people aged <18 years diagnosed with hypertension (including Secondary, Primary and White Coat Hypertension) by a dedicated Paediatric Hypertension service over a recent 6-year period.

		White coat	Primary	Secondary	*P* value
Total	*n* (% of total)	47 (15.0)	84 (26.8)	183 (58.2)	
Referral source	Primary care *n* (%)	3 (6.4)	3 (3.6)	2 (1.1)	<0.001
	Secondary care *n* (%)	25 (53.2)	58 (69.0)	65 (35.5)	
	Tertiary care *n* (%)	19 (40.4)	23 (27.4)	116 (63.4)	
Patients on antihypertensives on assessment	*n* (%)	3 (6.4)	39 (46.4)	139 (76)	<0.001
Sex	Male *n* (%)	15 (31.9)	39 (46.4)	87 (47.5)	0.158
	Female *n* (%)	32 (68.0)	45 (53.6)	96 (52.5)	
Age in years	Mean (SD)	13.4 (±3.4)^a^	14.2 (±2.5)^b^	8.9 (±6.1)^a,b^	<0.001^a,b^
Age category	<6 years *n* (%)	2 (4.3)	0 (0.0)^c^	72 (39.3)^d^	<0.001
	6–11 years *n* (%)	11 (23.4)	13 (15.5)	37 (20.2)	
	12–18 years *n* (%)	34 (72.3)	71 (84.5)^e^	74 (40.4)	
Ethnicity (*n* = 307)	White *n* (%)	26 (61.9)	48 (57.8)	146 (80.2)^f^	<0.001
	Black *n* (%)	7 (16.6)	25 (30.1)^g^	18 (9.9)	
	SE Asian *n* (%)	8 (19.0)	8 (9.6)	12 (6.6)	
	Other *n* (%)	1 (2.4)	2 (2.4)	6 (3.3)	
	Undisclosed *n* (%)	5	1	1	
Height	Mean (cm) (SD)	161.8 (±19.0)^a^	162.8 (±18.0)^b^	125.6 (±39.9)^a,b^	<0.001^a,b^
*Z* score height	Mean (SD)	1.25 (±1.88)^a^	0.64 (±1.62)^b^	-0.29 (±2.09)^a,b^	<0.001^a,b^
Weight	Mean (kg) (SD)	66.2 (±24.1)^a^	72.1 (±25.4)^b^	37.8 (±26.1)^a,b^	<0.001^a,b^
Z score weight	Mean (SD)	1.35 (±1.24)^a^	1.47 (±1.15)^b^	0.18 (±1.73)^a,b^	<0.001^a,b^
BMI	Mean (kg/m^2^) (SD)	24.6 (±6.5)^a^	26.6 (±7.3)^b^	20.3 (±5.4)^a,b^	<0.001^a,b^
*Z* score BMI	Mean (SD)	1.09 (±1.12)	1.32 (±0.97)^b^	0.49 (±0.97)^b^	<0.001^b^

^a^denotes significant difference in mean between white coat and secondary hypertensive groups.

^b^denotes significant difference in mean between primary and secondary hypertensive groups.

^c^denotes significantly lower than expected (*p* < 0.001).

^d^denotes significantly higher than expected (*p* < 0.001).

^e^denotes significantly higher than expected (*p* = 0.02).

^f^denotes significantly higher than expected (*p* = 0.009).

^g^denotes significantly higher than expected (*p* = 0.042).

### Primary and White Coat Hypertension

**Fig. 2:**
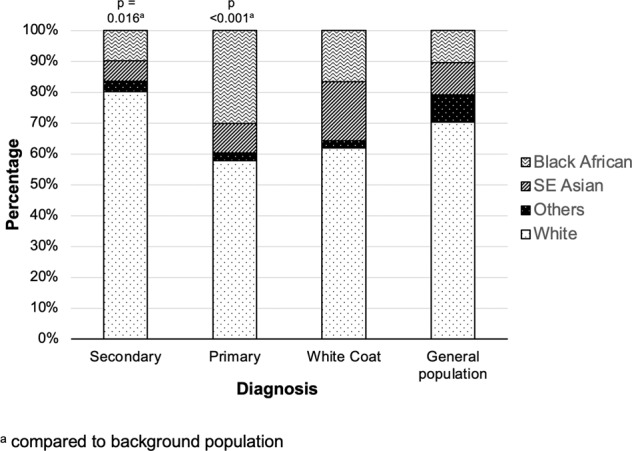
Distribution of hypertension phenotype by ethnicity compared to background population. Data includes patient stated ethnicity and pertains to those aged <18 years of age diagnosed with hypertension.

PH was diagnosed in 26.7% (*n* = 84) and WCH in 15.0% (*n* = 47) of those with hypertension. PH and WCH presented predominantly during adolescence (Fig. 1, *p* < 0.001). In patients under 6 years, there were no patients with PH and prevalence of WCH was 2.7% (*n* = 2/74) when home BP measurements were compared with office BP. There was a higher proportion of Black patients in the PH group compared to the background population (30% vs 11% [*p* < 0.001], Fig. 2).

### Secondary hypertension

Amongst those with hypertension, SH was diagnosed in 58.2% (*n* = 183), with SH due to renal disease in 50%, renovascular disease in 16%, cardiac in 10% and endocrine in 3% with other causes shown in Table 1A (Supplementary information). Children <6 years with hypertension were significantly more likely to have SH than PH (*p* < 0.001). Patients with SH presented throughout childhood, however the proportion of SH declined with increasing age category (Fig. 1). The ethnic distribution within each hypertensive subgroup is compared with the background population (Fig. 2). There were a higher proportion of white patients in the SH group compared to the background population (80% vs 70% [*p* = 0.02]).

### Body mass index

In patients with PH, BMI *z*-score was significantly higher compared to those with SH (1.32 ± 0.97 vs. 0.49 ± 0.97, *p* < 0.001) but not when compared with those with WCH (1.32 ± 0.97 vs. 1.09 ± 1.12, *p* = 0.65) or between sexes across all diagnostic groups (*p* = 0.24). In hypertensive patients, prevalence of excess weight was higher for PH and WCH compared to SH (56%, 53% and 36%, respectively [*p* = 0.02]). Amongst hypertensive children and adolescents <6 years there was a lower prevalence of excess weight compared with those ≥6 years (35% vs 49%, [*p* < 0.001]). In those with excess weight there was no difference in distribution of hypertension categories by ethnicity [*p* = 0.08].

## Discussion

We report data describing the demographics of a contemporary childhood hypertension population within a South of England paediatric hypertension service. The main finding is the high prevalence of PH in adolescence within our referral population: in hypertensive children over 12 years, there were similar proportions of PH and SH at 40% and 41%, respectively. There was also a surprising proportion of children in the 6–11 age group with PH and WCH at 21% and 18%, respectively (Fig. 1). Overall, 27% of hypertensive children and adolescents had PH but nearly 42% when both PH and WCH are considered together. Hypertension in children and adolescents has historically been assumed to be predominantly due to secondary causes. These first data from the UK suggests that whilst that remains true in the under 6 age group, for those over 6, PH and WCH must be considered in the differential diagnoses as an adolescent with hypertension now has an equal chance of being diagnosed with PH as SH.

These findings from the UK are in keeping with similar reports from Europe and the United States of America (USA), underscoring the increasing prevalence of PH in children and adolescents [6, 7]. A recent study from a hypertension service based in the USA identified PH as the predominant cause in 43% [17]. Hypertension services in China and Brazil have reported lower proportions of PH at 14–15% [18, 19]. These data likely reflect differences in referral practices and underlying population, although all reports highlight the predominant association with obesity [6, 7, 17–19]. In our population, we observed higher prevalence of excess weight in those with PH and WCH when compared with background childhood population; [20] and significantly higher BMI in those with PH compared to SH. We did not observe differences by ethnicity for those with excess weight but this may be limited because of small numbers of children and adolescents with hypertension from ethnic minorities. Our findings support the current consensus that excess weight is a significant risk factor for developing hypertension in childhood, further highlighting the importance of prevention and improved management of childhood obesity. Further, clinicians assessing an adolescent with raised BP may consider having a higher index of suspicion for secondary causes in those who are of normal weight.

Our data show a higher proportion of females at 58.2% of the total patients studied, 54% in PH and 68% in WCH respectively. Male predominance is well known in PH in adults, and this was also seen in a recent paediatric cohort with 56% male [17]. However, a smaller study from Brazil showed sex distribution more similar to ours with 58% females in their PH group. We also observed more females in the normotensive group (62%) so this may reflect referral patterns or an underlying factor specific to our regional population. Population level studies would be required to further investigate the true association between sex and childhood hypertension.

We observed an overrepresentation of non-White ethnicities diagnosed with PH and WCH when compared with the background population (Fig. 2). Similar findings have been reported previously in non-UK based population studies [21, 22]. Again, confirmation with UK based population studies are needed as our observations may be subjected to bias relating to referral patterns to our service. However, our findings suggest that children and adolescents from ethnic minorities presenting with raised BP are more likely to be diagnosed with PH than those of white ethnicity, which should be kept in mind by clinicians assessing and referring these children.

We observed that WCH has a similar patient phenotype to those with PH, presenting predominantly in adolescence and associated with increased BMI and in keeping with other recent reports [23]. WCH is not a benign phenomenon, with some patients progressing to hypertension in the future [24, 25]. It has been shown that uncontrolled WCH in adults increases risk for adverse cardiovascular outcomes [26, 27]. Long term outcomes for children and adolescents with WCH are unknown but data in paediatric patients suggests that these patients have increased prevalence of arterial stiffness and left ventricular hypertrophy compared to normotensive peers [21, 22].

An interesting finding from our report is that ~43% children referred for concern regarding high blood pressure were found to be normotensive following assessment by our service. Our assessment includes standardised auscultatory office BP measurements performed with the appropriate cuff size by a trained paediatric nurse when the child is settled. The high proportion of patients found to have normotension, especially the youngest, highlights the need for further training and adequate BP measurement equipment provision in non-specialist settings.

Out of office BP monitoring is essential in children to confirm hypertension, with 24-h ABPM well established as the gold standard method for performing out of office BP assessment [6, 28, 29]. ABPM is unfortunately not feasible in those aged <5-years due to the lack of normative data in this age group; yet 30% of patients referred to our service were <6 years. Our report highlights the utility of HDBPM to address this clinical need [15]. Intermittent BP monitoring at home using an oscillometric method of BP measurement has also been shown to have high reproducibility with office BP and daytime ambulatory BP [30, 31]. A key limitation of using this method is that currently there are no reference data for home BP measurement in this age group either through oscillometric or Doppler methods.

Overall 52% of our patients had been referred from Primary or Secondary Care; only a small proportion had previously undergone out of office BP monitoring to confirm hypertension. Despite this many had had extensive investigations to look for possible secondary causes of hypertension. Following referral to our service and evaluation of BP with us they had been found to be normotensive. As we did not formally collect data regarding this, we are unable to comment further but suspect this pattern may result in delays accessing an accurate assessment of BP level and treatment but also potentially unnecessary investigations. Ambulatory BP monitoring in children has been found to be cost saving within a tertiary paediatric setting but there are few data regarding its use in Secondary care [32, 33]. We suggest there is a need for improving provision of 24-hour ABPM in Secondary Care in the UK.

A major limitation to our findings relates to the single centre retrospective nature of our report and the inherent biases therein. Further, local referral patterns may influence our study population and this may also explain the relatively small numbers of children with PH for a large childhood population. Previous UK reports highlight inconsistency regarding measurement, interpretation and follow up of BP in children and adolescents within primary and secondary healthcare settings, and population cohort data from the USA confirms under-diagnosis of hypertension in the children and adolescents population [34, 35]. Overall, we suspect that many children and adolescents with elevated BP are either not recognised or not referred to our service and therefore cannot be considered as fully representative of the hypertensive children and adolescent population within the UK until other UK data are reported. We have also reported basic analyses of our data by ethnicity and BMI but recognise that the sample is relatively small to draw firm conclusions from these and that population cohort data would be required to investigate this further. Despite these limitations, to our knowledge this is the largest dataset from a dedicated hypertension service describing a contemporary population of children and adolescents with hypertension in the UK.

## Conclusion

Hypertensive children <6 years are most likely to have SH and negligible rates of WCH and PH. PH accounted for 27% of hypertension diagnoses in children and adolescents from our large paediatric hypertension service, with the highest prevalence in adolescence, those of non-White Ethnicity and with excess weight. Clinicians should consider rationalising investigations for secondary causes in those with the highest risk of PH.

### Summary

#### What is known about this topic

- Primary hypertension in children and young people is increasing in frequency.
- Primary hypertension is associated with excess weight.
- Primary hypertension may be more common in non white ethnic backgrounds.

#### What this study adds

- This is the first UK based report detailing demographics of children and young people evaluated in a dedicated hypertension clinic.
- Hypertensive adolescents assessed by our service have an equal likelihood of being diagnosed with primary hypertension vs. secondary hypertension.
- Excess weight and those from ethnic minority backgrounds were over-represented in young people diagnosed with primary hypertension.

## Supplementary information

Supplementary information

## Data Availability

The datasets generated during and/or analysed during the current study are available from the corresponding author on reasonable request.
