# Supplementary material for: Demographics of childhood hypertension in the UK: a report from the Southeast England
Source: J Hum Hypertens. 2022 Aug 6;37(7):554–9. doi: 10.1038/s41371-022-00732-7 (PMC10328827; doi:10.1038/s41371-022-00732-7)
Supplement: Supplementary file 1 — Supplementary information [file 41371_2022_732_MOESM1_ESM.docx]

**Figure 1A:** Out of office BP monitoring, pathway determining selection of modality

Office BP measured

≥5 years and able to tolerate APBM?

<5 years / not able to tolerate APBM?

Community nurse BP measurement

ABPM

HDBPM

Successful result

Unsuccessful

Unsuccessful

Footnote: BP: blood pressure; ABPM: ambulatory blood pressure monitoring; HDBPM: home doppler blood pressure monitoring

**Table 1A:** Secondary causes of hypertension (n=183)

| **Category** | **Diagnosis** | **Number** |
| --- | --- | --- |
| Renal | AKI | 2 |
|  | Congenital Abnormalities of the Kidney and Urinary Tract (CAKUT) | 11 |
| n = 92 | CKD (no cause given) | 11 |
| (50%) | Cystic kidney disease | 6 |
|  | Dysplasia | 6 |
|  | Glomerulonephritis | 4 |
|  | Haemolytic Uraemic Syndrome (HUS) | 2 |
|  | Nephrotic syndrome | 3 |
|  | Reflux nephropathy | 7 |
|  | Renal scarring | 9 |
|  | Renal transplant | 20 |
|  | Unknown | 11 |
|  |  |  |
| Renovascular  n = 26  (14%) |  | 26 |
|  |  |  |
| Cardiac | Coarctation | 10 |
| n = 18 | Congenital heart disease | 6 |
| (10%) | Other | 2 |
|  |  |  |
| Endocrine  n = 5  (3%) | Diabetes Mellitus / Insulin resistance | 5 |
|  |  |  |
| Drugs | Chemotherapy medication | 2 |
| n = 4  (2%) | ADHD medication  Steroid medication | 1  1 |
|  |  |  |
| Others | Neonatal insult | 11 |
| n = 38 | Neurological | 6 |
| (21%) | Syndrome | 9 |
|  | Neurofibromatosis and tuberous sclerosis | 4 |
|  | Hepatic | 1 |
|  | Unknown | 7 |
|  |  |  |
